# Associations between physical activity and prenatal depression and anxiety symptoms: a cross-sectional study

**DOI:** 10.3389/fpubh.2025.1666312

**Published:** 2025-12-05

**Authors:** Liang Mu, Hongli Yu, Guoping Qian

**Affiliations:** 1 Winter Olympic Academy, Harbin Sport University, Harbin, Heilongjiang, China; 2 College of Physical Education, Sichuan University of Science & Engineering, Zigong, Sichuan, China; 3 Department of Sport, Gdansk University of Physical Education and Sport, Gdansk, Poland

**Keywords:** prenatal mental health, maternal health promotion, public health strategy, non-pharmacological intervention, physical activity, depression, anxiety, pregnancy stages

## Abstract

Prenatal depression and anxiety pose a significant threat to maternal and neonatal health. Although physical activity (PA) improves mental wellbeing, its effects across pregnancy stages and relationship with sedentary behavior (SB) are not well understood. This study examined the relationships between PA, SB, and prenatal depression and anxiety, investigating differences throughout gestational stages. In this cross-sectional study, 501 pregnant women aged 18–45 completed the International Physical Activity Questionnaire - Short Form (IPAQ-SF), the Center for Epidemiologic Studies Depression Scale (CES-D), and the Generalized Anxiety Disorder-7 (GAD-7). Pearson correlations, linear regression, decision tree analysis, and one-way ANOVA were used to analyze associations and group differences. PA was inversely correlated with depression (*r* = −0.637, *p* < 0.01) and anxiety (*r* = −0.655, *p* < 0.01). Linear regression analysis demonstrated strong explanatory power for both depression (*R*^2^ = 0.839, adjusted *R*^2^ = 0.836) and anxiety (*R*^2^ = 0.844, adjusted *R*^2^ = 0.841), with overall significance (*p* < 0.001). Sedentary time and weekly MET-minutes were significant predictors (*p* < 0.001); increased sedentary time was associated with higher symptom scores, while greater PA was linked to lower depression and anxiety. Decision tree analysis identified a threshold of ≥2,346 MET-min/week as being associated with significantly reduced mental health symptom scores. One-way ANOVA revealed no differences in PA, depression, or anxiety across any stage of pregnancy. Higher levels of PA and reduced sedentary time were significantly associated with improved prenatal mental health. These associations were consistent across gestational stages, highlighting the importance of promoting sustained activity and reducing SB throughout pregnancy.

## 1 Introduction

Motherhood is widely recognized as a transformative and emotionally fulfilling life event (1). However, a significant proportion of pregnant women experience psychological distress during gestation (2). Hormonal fluctuations and evolving responsibilities contribute to the emergence of mental health disorders such as depression and anxiety, which pose significant threats to maternal wellbeing (3). These conditions, clinically characterized by persistent low mood, fatigue, excessive worry, sleep disturbances, and diminished engagement in daily activities (4, 5), have garnered increasing attention in global health discourses. Epidemiological data from the World Health Organization (WHO) indicates that approximately 10%–13% of expectant mothers worldwide exhibit depressive symptoms, while an even larger cohort (15%–20%) demonstrates anxious symptomatology (6, 7). Corresponding figures from the Centers for Disease Control and Prevention in the United States reveal prevalence rates of 14%–23% for antenatal depression and 10%–15% for anxiety disorders (8, 9). Comparable findings have been reported by the UK’s National Health Service, documenting moderate-to-severe psychological distress in approximately 12% of pregnant women (10).

Pregnancy can exacerbate psychological distress due to hormonal changes, physical discomfort, and stress related to future uncertainties (11). External stressors such as low socioeconomic status, inadequate social support, and unemployment demonstrate significant associations with increased risk of antenatal mental health disorders (12). These untreated conditions are linked to diminished daily functioning and elevated risks of adverse pregnancy outcomes including gestational hypertension, low birth weight, and preterm delivery (13). Maternal mental health also influences fetal neurodevelopment and infant behavioral regulation, with consequences often persisting into early childhood (14, 15). The management of antenatal mental health carries substantial public health implications extending beyond individual well-being. Epidemiological evidence reveals correlations between maternal anxiety and depression during pregnancy and increased incidence of behavioral and emotional disorders in offspring (15). Furthermore, untreated prenatal depression serves as a established predictor of postpartum depression, which may substantially impair mother-infant bonding and long-term family dynamics (16). These cascading effects highlight the necessity of developing accessible, scalable, and safe preventive interventions for implementation during pregnancy.

Physical activity (PA) has emerged as a significant low-cost, non-pharmacological intervention for mental health. Substantial evidence demonstrates its effectiveness in enhancing both physical and mental health outcomes (17, 18). The WHO recommends that all adults, including pregnant women, engage in at least 150 min of moderate-intensity aerobic PA weekly to maintain optimal health (19). Exercise physiologically increases neurotransmitter levels, particularly endorphins, serotonin, and dopamine, which play crucial roles in mood regulation and emotional resilience (20). Pregnancy, however, presents unique physical and emotional challenges that may influence an individual’s capacity and motivation to maintain activity levels. Nevertheless, moderate-intensity exercise during pregnancy demonstrates multiple health benefits, including reduced risk of gestational diabetes mellitus, improved cardiovascular function, and enhanced weight management (21). Notably, PA during pregnancy correlates with decreased anxiety and depressive symptoms. Empirical studies indicate that regular PA participation enhances body image perception, strengthens self-efficacy beliefs, improves social connectivity, and promotes effective emotional regulation (22). These psychosocial advantages hold particular significance during pregnancy, a period when many women experience heightened emotional vulnerability and sensitivity.

While the physical benefits of prenatal exercise are well-established, its psychological effects remain underexplored. Recent evidence indicates suboptimal PA levels among pregnant women (23). Existing literature from China and other regions has seldom examined the synergistic relationships between PA and sedentary behavior (SB) with antenatal depression and anxiety. Few studies have explored how these associations differ across gestational stages (24), despite recognized physiological and emotional transformations that may influence mental health trajectories (25). Elucidating these stage-specific dynamics is crucial for developing personalized non-pharmacological interventions to enhance maternal psychological wellbeing (26). Emerging research identifies SB as an independent risk factor for adverse psychological outcomes (27), necessitating differentiation between beneficial rest and prolonged inactivity during pregnancy (28). However, comprehensive investigations simultaneously evaluating both PA and SB across all trimesters remain scarce. The present study systematically investigates the associations of PA and SB with depressive and anxiety symptoms in pregnant women, while exploring potential variations across gestational stages. Through multivariable linear regression and decision-tree analyses incorporating sociodemographic and health-related covariates, we aim to establish a data-driven PA threshold associated with reduced psychological symptomatology, thereby enhancing clinical applicability. This research seeks to generate evidence-based recommendations for prenatal mental health management and inform trimester-specific targeted interventions. The findings may advance clinical and public health practices by improving maternal emotional wellbeing and pregnancy outcomes, while contributing to standardized prenatal PA guidelines.

## 2 Materials and methods

### 2.1 Study setting

This study was conducted in Nanjing, China, a major provincial capital with a large and diverse pregnant population, providing an appropriate setting to examine antenatal PA, SB, and mental health.

### 2.2 Study design

This study employed a cross-sectional quantitative survey design to investigate the associations between antenatal PA, SB, and symptoms of depression and anxiety among pregnant women. A cross-sectional approach was adopted to capture a single time-point snapshot across gestational stages and facilitate comparisons of these associations during the same calendar period.

### 2.3 Instruments

PA was assessed using the International Physical Activity Questionnaire-Short Form (IPAQ-SF), a globally validated instrument with established psychometric properties (24, 28). The standardized Chinese version employed in this study underwent rigorous translation and back-translation procedures following IPAQ protocols and has been previously validated in Chinese adult and pregnant populations (29). This tool systematically captures frequency and duration of PA across different intensity levels and SB during the preceding week. According to official scoring algorithms, raw data are converted into metabolic equivalent task (MET) minutes per week, where higher MET-min/week values indicate greater PA levels and extended sitting times reflect increased SB. The questionnaire’s measurement properties have been comprehensively validated across diverse populations including pregnancy cohorts (27), with Chinese validation studies demonstrating satisfactory test–retest reliability for this cultural adaptation. Depressive symptoms were evaluated using the Center for Epidemiologic Studies Depression Scale (CES-D), originally developed by Radloff (30, 31). The 20-item Chinese version requires participants to rate symptom frequency over the past week on a 4-point Likert scale (0 = rarely or none of the time to 3 = most or all of the time), yielding total scores ranging from 0–60. Elevated scores correspond to more severe depressive manifestations, with a cutoff score of ≥16 commonly applied in Chinese perinatal research to identify clinically significant depression (32). Extensive application in both clinical and community settings has demonstrated the CES-D’s strong psychometric characteristics among Chinese populations, consistently showing Cronbach’s *α* coefficients between 0.85 and 0.90 (33, 34), thereby confirming its excellent internal consistency, construct validity, and cultural appropriateness. The Generalized Anxiety Disorder-7 (GAD-7) scale (35, 36) was employed to assess anxiety symptoms. This validated 7-item screening instrument evaluates the severity of anxiety symptoms experienced over the preceding two-week period. The study utilized the validated Chinese version, which assigns scores ranging from 0 to 3 per item, yielding a total score range of 0–21. Higher total scores indicate greater anxiety severity, with cutoff values of 5, 10, and 15 corresponding to mild, moderate, and severe anxiety categories, respectively, (37). The GAD-7 demonstrates favorable psychometric properties including ease of administration, high internal consistency reliability, and established cross-cultural validity across diverse populations including pregnant women. Chinese validation studies have reported Cronbach’s *α* coefficients approaching 0.90 (38, 39).

The systematic integration of these Chinese-adapted and validated instruments ensured methodological soundness and measurement precision, thereby generating high-quality data to examine the complex interplay between PA, SB, and prenatal mental health outcomes.

### 2.4 Participants

Participants were recruited from maternal and child healthcare hospitals, community health service centers, and structured prenatal education programs. This multi-site recruitment strategy aimed to ensure an adequate sample size while enhancing the study’s external validity through diverse population representation across socioeconomic, occupational, and cultural dimensions. Data collection was conducted between March and May 2024, a timeframe selected to minimize temporal variability and facilitate systematic participant enrollment. Recruitment strategies included informational posters, community outreach initiatives, and collaboration with local healthcare providers and maternal support groups, which collectively maximized participation rates and ensured demographic heterogeneity within the study cohort.

Eligibility criteria were rigorously defined to ensure sample specificity and integrity. Inclusion required pregnant women aged 18–45 years regardless of gestational stage. Participants needed adequate cognitive and language proficiency to understand study protocols and independently complete self-administered questionnaires. Written informed consent was obtained prior to participation in accordance with institutional review board approval and established ethical research guidelines. Exclusion criteria included severe chronic medical conditions or diagnosed psychiatric disorders known to significantly affect emotional regulation or PA levels. Additional exclusion criteria comprised multiple pregnancies, medically prescribed bed rest, and recent exposure to major life stressors such as bereavement, marital separation, or acute family crises that could substantially compromise emotional stability. These parameters facilitated recruitment of a clinically homogeneous cohort appropriate for investigating associations between PA and mental health outcomes during pregnancy.

### 2.5 Sample size estimation

The required sample size for this study was prospectively calculated using G*Power 3.1 software based on standard practices in behavioral and public health research. The *a priori* power analysis specified a small effect size (Cohen’s *d* = 0.2), reflecting common effect sizes in studies of PA and mental health outcomes (e.g., depression, anxiety) during pregnancy. To maintain statistical rigor and control Type I error rate, the significance level (*α*) was established at 0.01 with a target power level (1–*β*) of 0.95, ensuring 95% probability of detecting true effects. Under these parameters, G*Power calculations indicated that a minimum sample of 464 participants would achieve sufficient statistical power for planned analyses involving Pearson correlations and multivariable linear regression models. Considering potential methodological challenges including non-response, missing data, and participant attrition, the target sample size was conservatively inflated to 501 pregnant women. This adjustment accounted for anticipated data loss while maintaining adequate statistical power throughout fieldwork operations. The actual achieved sample size exceeded this minimum requirement, thereby enhancing both the reliability of statistical inferences and the external validity of findings within prenatal mental health research.

### 2.6 Data collection

To enhance temporal consistency and ensure data reliability, all participants were recruited and surveyed during a single calendar period (March–May 2024). This approach mitigated potential confounding factors such as seasonal variations, temporal misalignment, or extraneous health events. Data collection followed a standardized protocol utilizing validated self-report instruments to assess key study variables. Trained research personnel screened prospective participants against predetermined eligibility criteria and extended participation invitations to qualified individuals. After receiving comprehensive information regarding the study’s objectives, methodology, and voluntary withdrawal rights, participants provided electronic informed consent. Online data collection was conducted through the Wenjuanxing (Questionnaire Star) platform, which incorporated automated safeguards preventing duplicate submissions of identical standardized questionnaires. Completeness of responses was verified prior to secure storage in encrypted, password-protected databases accessible exclusively to authorized research personnel. The study received ethical approval from the Harbin Institute of Sports Ethics Committee (Approval No: 2024057; Date: 20 February 2024), in accordance with established ethical guidelines for research involving human subjects. To optimize population representativeness and minimize selection bias, participants were systematically recruited through stratified random sampling across maternity and child health hospitals, community health service centers, and prenatal education programs. Data acquisition procedures were implemented in controlled, supervised environments to maintain methodological rigor and facilitate participant comprehension. Dual independent verification of exported datasets by separate research team members effectively minimized transcription and processing errors while ensuring data accuracy.

### 2.7 Variables

The primary outcome variables in this study comprised PA levels, symptoms of depression, and symptoms of anxiety. To control for potential confounding factors, comprehensive demographic, socioeconomic, and health-related covariates were systematically collected. Demographic data encompassed chronological age (measured in years), educational attainment (categorized using ordinal ranking), occupational status (classified as employed, unemployed, or other), and annual household income. Income was stratified into four categories: low (<30,000 CNY/year), lower-middle (30,000–60,000 CNY/year), upper-middle (60,000–120,000 CNY/year), and high (>120,000 CNY/year). Health-related covariates comprised pre-pregnancy weight and height [used to calculate body mass index (BMI; kg/m^2^)], gestational trimester, and parity (primigravida or multigravida). Medical history data included documentation of chronic illnesses or pre-existing psychological conditions. These covariates were incorporated into multivariate statistical models to reduce confounding and increase effect estimates precision and validity.

### 2.8 Data analysis and quality control

All analyses were conducted in IBM SPSS Statistics using two-tailed tests with *α* = 0.05. Continuous variables are presented as mean ± SD with 95% confidence intervals (CIs), and categorical variables as frequencies and percentages. To mitigate bias from missing data, multiple imputation was applied before inferential analyses. Bivariate associations among PA (MET-min/week), sedentary time (min/day), depressive symptoms (CES-D), anxiety symptoms (GAD-7), and covariates were examined using Pearson correlations. Adjusted associations were estimated with multivariable linear regression models using CES-D and GAD-7 total scores as dependent variables and including PA and sedentary time as primary predictors, with covariates (BMI, age, education, employment status, parity, and household income). Collinearity was evaluated via variance inflation factors (VIF) and tolerance. Model assumptions and robustness were formally checked. Residual autocorrelation was assessed with the Durbin–Watson statistic; when mild positive autocorrelation was indicated, heteroskedasticity- and autocorrelation-robust (Newey-West) standard errors were computed, and sensitivity models were re-estimated using generalized least squares with an AR(1) error structure. To compare outcomes across gestational stages (first ≤13 weeks; second 14–27 weeks; third ≥28 weeks), one-way ANOVA was performed for PA, sedentary time, CES-D, and GAD-7, with Levene’s test used to confirm homogeneity of variances. Finally, we used a decision tree analysis to find a data-driven PA threshold that best separated people with more psychological symptoms from those with fewer symptoms. This gave us an interpretable risk stratification that worked well with the regression results.

## 3 Results

### 3.1 Descriptive statistics

Table 1 shows the basic information about the 501 pregnant participants. The average age was 31.02 ± 3.42 years (95% CI: 30.72, 31.32), which means that most of the people in the group were in their early thirties. The average BMI was 25.60 ± 2.75 kg/m^2^ (95% CI: 25.36, 25.84), which is in the range of being overweight according to the WHO. The average amount of time spent sitting still was 274.72 ± 235.69 min per day (95% CI: 254.03, 295.41), which is about 4.6 h. This shows that this level of inactivity every day could be negative for your health. On the other hand, the average PA levels were very high at 9546.05 ± 11691.52 MET-minutes/week (95% CI: 8519.80, 10572.30), which is much higher than the WHO’s minimum recommendations. While encouraging, these results may reflect an overestimation due to self-reporting via the IPAQ-SF. Mental health scores indicated elevated symptomatology, with mean CES-D at 25.39 ± 11.60 (95% CI: 24.38, 26.40) and GAD-7 at 8.14 ± 7.42 (95% CI: 7.49, 8.80), suggesting clinically relevant psychological distress. 62.07% had a bachelor’s degree or higher, and 47.11% were working full-time. There are issues to consider about when it comes to SB and stress at work. The majority of participants (82.64%) were at or before 26 weeks of gestation, and the parity was nearly equal (46.71% primigravida). Overall, the cohort was an excellent way for investigating at how prenatal PA and mental health affect each other.

**Table 1:** Descriptive statistics of key study variables.

Variables	Mean ± SD	Mean 95% CI
Height (cm)	159.38 ± 2.16	159.19, 159.57
Weight (kg)	65.16 ± 6.30	64.61, 65.71
BMI (kg/m^2^)	25.60 ± 2.75	25.36, 25.84
Age (years)	31.02 ± 3.42	30.72, 31.32
Physical activity (MET-min/week)	9546.05 ± 11691.52	8519.80, 10572.30
Sedentary time (min/day)	274.72 ± 235.69	254.03, 295.41
Total depression score	25.39 ± 11.60	24.38, 26.40
Total anxiety score	8.14 ± 7.42	7.49, 8.80

SD, standard deviation; 95% CI, 95% confidence interval; kg, kilogram; kg/m^2^, kilograms per square meter; MET, metabolic equivalent of task; min, minutes.

### 3.2 Correlation analysis

Table 2 shows the Pearson correlation coefficients among significant variables: PA (MET-minutes), sedentary time, CES-D, GAD-7, and specific demographics. PA was significantly inversely correlated with CES-D (*r* = −0.637, *p* < 0.01) and GAD-7 (*r* = −0.655, *p* < 0.01), indicating that higher activity levels correspond with reduced symptom scores. Conversely, sedentary time exhibited robust positive correlations with CES-D (*r* = 0.901, *p* < 0.01) and GAD-7 (*r* = 0.899, *p* < 0.01), signifying that extended inactivity is associated with detrimental mental health outcomes. MET-minutes were positively associated with weight, BMI, age, and parity but negatively associated with education, employment, and income, implying that women with higher socioeconomic status may engage in more SB, possibly due to occupational demands. CES-D and GAD-7 were also positively related to sedentary time, income, and employment status but were inversely related to BMI and parity. These correlations support associations of higher PA with lower symptom burden and of greater sedentary time with higher symptom burden during pregnancy. They also point out the importance of targeted interventions.

**Table 2:** Pearson correlation coefficients among study variables.

Variables	MET-minutes	Depression score	Anxiety score
Height	−0.033	0.037	0.052
Weight	0.463**	−0.191**	−0.211**
BMI	0.417**	−0.140**	−0.166**
Age	0.291**	−0.038	−0.047
Education level	−0.457**	0.261**	0.270**
Employment status	−0.489**	0.309**	0.322**
Gestational stage	0.013	−0.065	−0.064
Sedentary time	−0.562**	0.901**	0.899**
Parity	0.136**	−0.137**	−0.132**
Smoking/drinking	−0.072	0.083	0.055
Chronic diseases	−0.042	0.069	0.063
Household income	−0.459**	0.299**	0.312**
Pregnancy health	0.000	0.000	0.000
Psychological history	−0.047	0.058	0.060
Delivery mode	0.045	−0.044	−0.038
Depression score—MET-minutes (−0.637**)		Anxiety score—MET-minutes (−0.655**)	

**Significant (at *p* < 0.01); *significant (at *p* < 0.05).

### 3.3 Multivariable linear regression analysis

Table 3 summarizes the multivariable linear regression results for predictors of prenatal depressive symptoms. The model explained a large proportion of variance in CES-D scores (*R*^2^ = 0.839; adjusted *R*^2^ = 0.836). Sedentary time was the strongest positive predictor (*B* = 0.039, *p* < 0.001), with each additional sedentary minute per day associated with a 0.039-point higher CES-D score. PA (MET-min/week) showed an inverse association (*B* ≈ −0.000, *p* < 0.001), reflecting its fine unit scale. Parity was modestly protective (*B* = −0.711, *p* = 0.050), while BMI, age, education, employment, and income were nonsignificant. Multicollinearity was minimal (VIF < 2.6). Despite some residual dependence (DW = 1.088), results were robust across alternative estimations [Newey-West; GLS with AR (1), *ϕ* = 0.307] (Supplementary Table S1). Overall, sedentary time emerged as a key modifiable correlate, whereas higher PA related to fewer depressive symptoms.

**Table 3:** Multivariable linear regression analysis of depression scores (CES-D).

Variables	Unstandardized coefficients		Standardized coefficients	*t*	*p*	Collinearity diagnosis	
	*B*	*SE*	*Beta*			VIF	Tolerance
Constant	17.857	3.218	—	5.549	0.000**	—	—
BMI	−0.011	0.087	−0.003	−0.127	0.899	1.304	0.767
Age	−0.028	0.068	−0.008	−0.413	0.680	1.216	0.822
Educational level	−0.238	0.265	−0.019	−0.898	0.370	1.367	0.732
Employment status	0.032	0.245	0.003	0.131	0.896	1.465	0.683
Sedentary time	0.039	0.001	0.796	34.250	0.000**	1.648	0.607
Parity	−0.711	0.362	−0.037	−1.966	0.050*	1.073	0.932
Household income level	0.493	0.299	0.035	1.647	0.100	1.360	0.735
MET-minutes	−0.000	0.000	−0.173	−5.927	0.000**	2.586	0.387
*R^2^*	0.839						
Adjusted *R^2^*	0.836						
*F*	*F* (8,492) = 319.921, *p* < 0.001						
*D-W value*	1.088						

Dependent variable = total depression score. *B*, unstandardized regression coefficient; SE, standard error; *β* (Beta), standardized regression coefficient; t, t-statistic; *p*, *p*-value; VIF, variance inflation factor; BMI, body mass index (kg/m^2^); MET, metabolic equivalent of task; *R*^2^, coefficient of determination; *F*, F-statistic; D–W, Durbin–Watson statistic. **Significant (at *p* < 0.01); *significant (at *p* < 0.05).

Table 4 presents multivariable regression analyses identifying predictors of prenatal anxiety. The model demonstrated robust explanatory power (*R*^2^ = 0.844; adjusted *R*^2^ = 0.841), accounting for approximately 84% of variance in GAD-7 scores. Sedentary time emerged as the strongest positive predictor (*B* = 0.025, *p* < 0.001), indicating that prolonged daily inactivity significantly increased anxiety likelihood. PA expressed in MET-minutes per week showed a significant inverse association with anxiety symptoms (*B* ≈ −0.000, *p* < 0.001), reflecting the precision of its unit scale measurement. Non-significant covariates included BMI, age, education, employment status, parity, and income. Multicollinearity diagnostics confirmed tolerance (all VIF < 2.6), and robust estimation methods (Newey-West correction; generalized least squares with AR (1) correlation structure, *ϕ* = 0.290) yielded consistent parameter estimates (Supplementary Table S2). These findings corroborate sedentary time’s positive association and PA’s negative association with prenatal anxiety (both *p* < 0.001). The results establish SB and PA as consistent behavioral markers of antenatal anxiety, emphasizing the critical role of movement-based interventions in perinatal mental health management.

**Table 4:** Multivariable linear regression analysis of anxiety scores (GAD-7).

Variables	Unstandardized coefficients		Standardized coefficients	*t*	*p*	Collinearity diagnosis	
	*B*	*SE*	*Beta*			VIF	Tolerance
Constant	4.577	2.028	—	2.257	0.024*	—	—
BMI	−0.063	0.055	−0.023	−1.153	0.249	1.304	0.767
Age	−0.018	0.043	−0.008	−0.411	0.681	1.216	0.822
Educational level	−0.164	0.167	−0.021	−0.984	0.326	1.367	0.732
Employment status	0.059	0.154	0.008	0.382	0.703	1.465	0.683
Sedentary time	0.025	0.001	0.784	34.234	0.000**	1.648	0.607
Parity	−0.332	0.228	−0.027	−1.456	0.146	1.073	0.932
Household income level	0.351	0.189	0.039	1.860	0.063	1.360	0.735
MET-minutes	−0.000	0.000	−0.187	−6.516	0.000**	2.586	0.387
*R^2^*	0.844						
Adjusted *R^2^*	0.841						
*F*	*F* (8,492) = 331.700, *p* < 0.001						
*D-W value*	1.207						

Dependent variable = total anxiety score. *B*, unstandardized regression coefficient; SE, standard error; *β* (Beta), standardized regression coefficient; *t*, *t*-statistic; *p*, *p*-value; VIF, variance inflation factor; BMI, body mass index (kg/m^2^); MET, metabolic equivalent of task; *R*^2^, coefficient of determination; *F*, F-statistic; D–W, Durbin–Watson statistic. **Significant (at *p* < 0.01); *significant (at *p* < 0.05).

### 3.4 Decision-tree analysis

Table 5 presents the decision-tree analysis revealing that approximately 2,346 MET-min/week serves as a threshold distinguishing between elevated and reduced psychological symptom levels among pregnant women. Participants meeting or exceeding this threshold (*n* = 283) demonstrated significantly lower mean GAD-7 (1.77) and CES-D (15.47) scores, approaching or falling below established screening cut-off values. Conversely, those below the threshold (*n* = 218) demonstrated considerably higher mean scores (GAD-7 = 16.41; CES-D = 38.28). This pattern is consistent with the regression analysis results, indicating that during pregnancy, increased PA is positively associated with better mental health outcomes. The identified threshold should be regarded as exploratory, requiring independent validation before clinical or practical application.

**Table 5:** Decision-tree–derived physical activity threshold and group differences in CES-D and GAD-7.

Node	Splitting condition	*n*	Depression score	Anxiety score	Node	Splitting condition
			Deviance	yval	Deviance	yval
1	Root node	501	27531.65	8.143713	67273.54	25.39321
2	MET_minutes ≥2,346	283	359.5265	1.773852	1400.495	15.46996
3	MET_minutes <2,346	218	782.844	16.41284	1829.486	38.27523

### 3.5 One-way ANOVA across gestational stages

Table 6 presents the one-way ANOVA comparing PA, sedentary time, depression (CES-D), and anxiety (GAD-7) across trimesters. No between-group differences were observed for PA (*F* = 0.0887, *p* = 0.7659), anxiety (GAD-7) (*F* = 2.055, *p* = 0.1523), or depression (CES-D) (*F* = 2.1002, *p* = 0.1479); sedentary time likewise showed no detectable differences. Levene’s tests were non-significant for all outcomes (all *p* > 0.05) (Supplementary Table S3), supporting homogeneity of variances. Overall, mean PA/SB and symptom levels appeared stable across trimesters in this sample, consistent with broadly similar associations between PA/sedentary time and mental health irrespective of trimester.

**Table 6:** One-way ANOVA results comparing physical activity, sedentary time, CES-D, and GAD-7 across pregnancy trimesters.

Trimester	*n*	Physical activity (MET min/week)	Sedentary time (min/day)	Depression (CES D)	Anxiety (GAD 7)
1st	206	8977.03 ± 11871.05	285.23 ± 235.19	26.26 ± 11.74	8.69 ± 7.56
2nd	208	10400.49 ± 11850.20	274.06 ± 235.55	24.99 ± 11.60	7.89 ± 7.33
3rd	87	8850.60 ± 10851.95	251.38 ± 238.22	24.30 ± 11.25	7.45 ± 7.29

MET, metabolic equivalent of task; CES-D, Center for Epidemiologic Studies Depression Scale; GAD-7, Generalized Anxiety Disorder-7 scale.

## 4 Discussion

Pregnant women face substantial mental health challenges, with depressive and anxiety symptoms among the most prevalent. In this study, higher PA was associated with lower CES-D and GAD-7 scores. In contrast, greater SB was associated with higher psychological-symptom burden. Based on the descriptive data, participants in this study reported a mean CES-D score of 25.39 ± 11.60 (95% CI: 24.38–26.40) and a mean GAD-7 score of 8.14 ± 7.42 (95% CI: 7.49–8.80), consistent with an elevated average symptom burden in this antenatal cohort (30–32). Considering that CES-D scores ≥16 and GAD-7 scores ≥5 represent established screening thresholds, these cutoff values indicate that a significant proportion of women exhibited clinically meaningful psychological distress (31). When interpreting the observed associations between PA, SB, and mental health outcomes within this cohort, these contextual considerations warrant careful attention (23). Interestingly, we observed no between-trimester differences in mean PA or symptom levels. These findings suggest that promoting PA while reducing SB could represent effective non-pharmacological interventions for enhancing prenatal mental health, pending confirmation through longitudinal and interventional studies. These insights may inform the development of continuous prenatal mental healthcare frameworks by offering practical approaches to mitigate psychological distress during pregnancy and influence public health policy formulation. Consistent with prior research (40), our study demonstrates PA’s positive association with pregnant women’s mental health, revealing inverse relationships between PA levels and depressive/anxiety symptoms, alongside direct correlations between sedentary time and psychological symptomatology. The observed detrimental effects of prolonged sitting on mental wellbeing might stem from metabolic dysregulation and systemic inflammation promotion (41), thereby highlighting PA’s potential as an accessible, non-pharmacological intervention for perinatal mental health management.

We observed positive associations between PA and anthropometric indicators including weight, body mass index (BMI), parity, and maternal age. These correlations suggest that physiological adaptations and lifestyle modifications during pregnancy may facilitate increased PA levels. In contrast, SB demonstrated significant positive associations with higher educational attainment, full-time employment status, and household income levels. This sociodemographic pattern aligns with existing literature examining occupational SB patterns and their mental health implications (42). Our analysis revealed no significant differences in PA-related mental health benefits across trimesters, a finding consistent with previous longitudinal investigations (43). While accumulating evidence supports PA’s protective effects against perinatal psychological morbidity, prior studies have not systematically examined gestational stage-specific variations in these associations. Our results substantiate continuous mental health benefits of PA throughout pregnancy, reinforcing clinical recommendations for maintaining moderate-intensity exercise across all gestational phases. Longitudinal analyses demonstrate typical PA trajectories peak during mid-pregnancy before declining in later stages due to progressive physical constraints (44). These temporal variations likely result from multifactorial influences including social support networks, medical counseling, and individual adaptive capacities. Targeted interventions are therefore essential to support sustained PA during late pregnancy when women often require additional assistance.

PA may promote mental health through multiple biological mechanisms, including endorphin release and regulation of key neurotransmitter systems (45). Endorphins, the body’s endogenous opioid peptides, play crucial roles in enhancing mood and alleviating pain perception. Moderate-intensity exercises such as brisk walking or light jogging demonstrate particular efficacy in stimulating endorphin secretion (46), thereby improving psychological well-being while reducing anxiety and depressive symptoms. The physiological elevation of endorphin levels during pregnancy facilitates pain modulation during labor (47), an effect that can be amplified through moderate-intensity exercise which concurrently stabilizes emotional tone (48). Additionally, PA exerts mood-enhancing effects via upregulation of serotonin and dopamine signaling pathways (49). Exercise increases serotonin biosynthesis while attenuating its metabolic degradation, resulting in improved affective regulation, sleep quality, and reduced depressive symptomatology (50). The activation of dopaminergic pathways during PA engages the brain’s reward circuitry, promoting enhanced motivational drive, pleasure perception, and life satisfaction. These coordinated neurochemical adaptations collectively contribute to improved emotion regulation capacity and stress resilience (51). PA plays a critical role in regulating the hypothalamic–pituitary–adrenal (HPA) axis, which governs the body’s stress response mechanisms (52). Cortisol, a hormone indispensable for maintaining pregnancy and fetal development, undergoes physiological elevation during gestation; however, chronically elevated concentrations may compromise maternal and fetal wellbeing (53, 54). Moderate aerobic exercise maintains stable cortisol concentrations, thereby stabilizing mood fluctuations and reducing chronic stress exposure (55). PA stimulates the release of neurotrophic factors from the brain that enhance cognitive function and neural plasticity (56, 57). These neurotrophic factors are particularly crucial for facilitating maternal physiological adaptation during gestation and supporting fetal neurodevelopment (58, 59). Exercise training enhances brain-derived neurotrophic factor production, facilitates emotional regulation, promotes neuronal growth, and modulates cortisol homeostasis through HPAA regulation (60). This neurobiological mechanism strengthens psychological resilience while alleviating anxiety and depressive symptomatology (61).

In addition to its physical effects, PA improves psychological health through enhanced self-efficacy, distraction from negative thoughts, and improved body image (62, 63). Increased self-efficacy - the belief in one’s capacity to overcome challenges - contributes to emotional stability and resilience (64). Regular exercise promotes feelings of accomplishment and perceived control, which may enhance maternal confidence and facilitate postpartum recovery. Specifically, yoga and brisk walking enable psychological detachment from stressors while reducing rumination. These activities provide cognitive resets that help regulate mood and mitigate anxiety. Furthermore, PA positively influences body image, which frequently undergoes significant changes during pregnancy (65). Given the high prevalence of body dissatisfaction during this period (66), low-intensity exercises and yoga practices can enhance bodily awareness and acceptance, thereby promoting psychophysical harmony. Conversely, prolonged SB adversely affects metabolic function, inflammatory processes, and hormonal balance, potentially exacerbating mental health issues. Extended sitting periods reduce energy expenditure, exacerbate insulin resistance, and promote dysglycemia, all of which may intensify anxiety and depressive symptoms. Chronic inactivity also induces systemic low-grade inflammation that disrupts neurochemical pathways implicated in mood regulation. Additionally, sedentary lifestyles correlate with elevated cortisol secretion (67), establishing a pathological cycle involving heightened stress responses and inflammatory activation (68). These interrelated mechanisms collectively amplify physiological and psychological stress burdens during pregnancy. Our findings suggest that moderate PA modulates these pathways by improving metabolic and inflammatory markers while safeguarding maternal and fetal wellbeing (69). SB may exacerbate psychological stress through disruption of sleep architecture and dietary quality. Prolonged inactivity has been shown to disturb circadian rhythmicity and sleep continuity, both critical for psychological homeostasis (70). Moreover, SB demonstrates strong associations with suboptimal dietary intake, which negatively affects neurotransmitter balance and emotional regulation. Beyond these behavioral pathways, multiple unmeasured psychosocial and lifestyle variables likely simultaneously influence PA levels and sedentary duration in this investigation (71). For example, compromised sleep quality during gestation could simultaneously reduce daily movement and intensify anxiety/depressive symptomatology, thereby artificially strengthening observed PA-mental health relationships (72). Similarly, individuals perceiving insufficient social support might increase time spent in sedentary pursuits within domestic settings, heightening affective vulnerability (73). Dietary practices characteristically accompanying sedentary lifestyles may worsen mood instability through metabolic or inflammatory pathways (74). Given the absence of direct measurements for these covariates, residual confounding cannot be entirely excluded from consideration.

This study underscores the critical role of PA in prenatal mental health management and offers actionable recommendations for clinical practice and public health policy. Our findings demonstrate that higher PA levels significantly reduce depressive and anxiety symptoms, while prolonged SB exacerbates these conditions. These results emphasize the importance of promoting moderate-intensity PA and minimizing SB during pregnancy. For individuals maintaining prolonged sitting patterns, such as full-time workers or stay-at-home mothers, integrating brief, frequent activity breaks into daily schedules may effectively reduce sedentary time. High-risk individuals with severe depression or anxiety symptoms should receive personalized, stage-appropriate exercise guidance to ensure safety and optimize benefits. The absence of significant differences across trimester-specific analyses suggests these interventions can be safely implemented throughout gestation. Decision tree analysis further substantiates this by identifying specific MET-minute thresholds associated with reduced mental health risks. Notably, our data-driven analysis identified 2,346 MET-minutes/week as a reference value indicating improved mental health outcomes at higher activity levels. However, this threshold must be contextualized within clinical safety parameters, as self-reported PA data from IPAQ-SF typically overestimate actual activity levels by up to 84% compared to objective measurements (75). Consequently, while this threshold provides valuable insights into dose–response relationships between PA volume and prenatal mental health, it should not serve as a universal clinical guideline. Current WHO guidelines recommending ≥150 min/week of moderate-intensity aerobic PA for pregnant women remain appropriate for substantial health benefits. They may gradually make their way up to 300 min a week as they feel comfortable (76). Other professional groups have also made similar suggestions. For example, the 2019 Canadian guideline for PA throughout pregnancy and the ACOG Committee Opinion No. 650 both say that women should do 150 min of moderate-intensity activity per week, with adjustments made based on their health and stage of pregnancy (77–79). Our data indicate that elevated activity levels correlate with diminished symptoms of anxiety and depression; however, clinical recommendations must be tailored to each woman’s health status, physical abilities, and stage of pregnancy.

Low-impact activities are especially beneficial for pregnant women in the third trimester as it reduce the risk of injury while providing health benefits without putting too much pressure on joints or increasing physical discomfort (40, 78, 80). For individuals with low baseline activity levels, gradual progression of PA intensity is recommended over abrupt transitions to excessive exertion, thereby minimizing fatigue-related risks (79). Implementation of individualized PA interventions requires concurrent provision of personalized education regarding appropriate exercise intensities to optimize safety and efficacy. Continuous biopsychosocial monitoring combined with dynamic adjustment of activity parameters based on real-time symptom reporting constitutes essential components of safe prenatal exercise programming. Given the inherent variability in maternal physiological responses, healthcare providers must individualize PA prescriptions to maintain mental health benefits while eliminating potential threats to maternal-fetal wellbeing. The high-activity threshold defined in this investigation serves as a reference standard requiring clinical adaptation to accommodate heterogeneous physiological demands across diverse pregnant populations. Public health strategies should prioritize development of scalable, culturally sensitive frameworks promoting sustainable PA engagement throughout gestation, ensuring equitable access for all women regardless of socioeconomic background or gestational stage. Multilevel community interventions integrating evidence-based educational components demonstrate capacity to reduce SB-associated pathologies while simultaneously improving perinatal mental health trajectories and longitudinal maternal-child health outcomes.

Based on these findings, we suggest that healthcare organizations, government agencies, and communities work together to include prenatal PA promotion in public health education programs. During regular prenatal visits, healthcare providers can suggest specific activities and talk about the dangers of being inactive for long periods of time. Community-based programs that make exercise easy to get to can create supportive spaces that encourage people to keep doing moderate PA. These strategies can work together to improve the mental health of the mother, the development of the fetus, and the outcome of the pregnancy.

### 4.1 Study limitations

This study has several limitations. First, both PA and mental-health outcomes were self-reported (IPAQ-SF, CES-D, GAD-7), which are susceptible to recall and social-desirability bias; notably, IPAQ-SF may overestimate activity (up to ~84% versus device measures), potentially inflating associations (75). Second, the cross-sectional design precludes causal inference and tracking change across pregnancy; longitudinal designs are needed. Third, recruitment from a single city may limit generalizability across cultural, social, and healthcare contexts. The data-driven decision-tree split (≥2,346 MET-min/week) is context- and instrument-dependent and should not be treated as prescriptive. Fourth, the threshold was derived and evaluated within the same dataset and therefore remains exploratory, requiring external validation in multi-center, longitudinal, or international cohorts before informing programs or clinical decision-support. Fifth, several potentially important covariates-such as sleep quality, perceived social support, dietary habits, and clinician advice regarding PA-were not systematically measured, so residual confounding is possible (e.g., poor sleep could both reduce PA and increase anxiety). Finally, specific obstetric contraindications to exercise (e.g., placenta previa, hypertensive complications) were not systematically gathered; therefore, the application of these findings should be conducted within individualized, clinician-directed risk–benefit evaluations. Subsequent research ought to integrate device-based PA assessments, a wider array of psychosocial and lifestyle determinants, and multicenter sampling to improve validity and generalizability.

## 5 Conclusion

This cross-sectional study explored association between PA and depressive/anxiety symptoms in pregnant women, identifying relationships between activity levels and prenatal mental health. The findings indicate that higher PA is associated with lower CES-D and GAD-7 scores, whereas greater SB was associated with higher symptom levels. These associations were observed across trimesters in this sample, however, equality of associations by trimester was not established without formal moderation tests. Maintaining PA and limiting SB may be relevant throughout gestation when medically appropriate and absent contraindications. Public health and antenatal services may consider integrating brief, trimester-appropriate counseling that encourages moderate PA and reduces prolonged sitting, especially for pregnant women who screen positive for psychological distress. Limitations include the cross-sectional design and reliance on self-reported measures; therefore, the observed associations should be confirmed in longitudinal and intervention studies, preferably with device-based assessment of PA and SB.

## Data Availability

The raw data supporting the conclusions of this article will be made available by the authors, without undue reservation.
