# Supplementary material for: Associations between physical activity and prenatal depression and anxiety symptoms: a cross-sectional study
Source: Front Public Health. 2025 Dec 5;13:1666312. doi: 10.3389/fpubh.2025.1666312 (PMC12714648; doi:10.3389/fpubh.2025.1666312)
Supplement: Supplementary file 1 [file Table_1.docx]

# Supplementary Table 1. Correlates of prenatal depressive symptoms (CES-D): multivariable linear regression

| **Predictor** | **B (SE)** | **95% CI** | **t** | **p** |
| --- | --- | --- | --- | --- |
| Intercept | 17.603 (3.227) | 11.278 to 23.928 | 5.455 | <0.001 |
| PA (MET total) | −0.000212 (0.000032) | −0.000275 to −0.000149 | −6.6263 | <0.001 |
| Sedentary time (min) | 0.036744 (0.001365) | 0.034069 to 0.039419 | 26.919 | <0.001 |
| BMI | 0.006 (0.084) | −0.158 to 0.170 | 0.0733 | 0.942 |
| Age | −0.036 (0.071) | −0.176 to 0.103 | −0.5086 | 0.611 |
| Education | −0.131 (0.248) | −0.618 to 0.355 | −0.5283 | 0.598 |
| Employment status | −0.014 (0.232) | −0.470 to 0.442 | −0.0613 | 0.951 |
| Household income level | 0.530 (0.279) | −0.017 to 1.077 | 1.9018 | 0.058 |
| Parity | −0.483 (0.398) | −1.264 to 0.298 | −1.2130 | 0.226 |

**Scaled interpretation.** ΔCES-D per **+60 min** sedentary = **+2.20** (95% CI **+2.04 to +2.37**); ΔCES-D per **+100 MET-min** PA = **−0.021** (95% CI **−0.027 to −0.015**).

**Model diagnostics.** AIC = **2985.51**; BIC = **3031.69**; logLik = **−1481.76**; Residual SE = **4.74** (df = 492); GLS-AR(1) ϕ = **0.307**; all VIF < 2.6.

*Note: MET = metabolic equivalent of task; BMI = body mass index (kg/m²); CI = confidence interval. Unstandardized coefficients. Positive B indicates higher symptom scores.*
