# Supplementary material for: Associations between physical activity and prenatal depression and anxiety symptoms: a cross-sectional study
Source: Front Public Health. 2025 Dec 5;13:1666312. doi: 10.3389/fpubh.2025.1666312 (PMC12714648; doi:10.3389/fpubh.2025.1666312)
Supplement: Supplementary file 2 [file Table_2.docx]

# Supplementary Table 2. Correlates of prenatal anxiety (GAD-7): multivariable linear regression

| **Predictor** | **B (SE)** | **95% CI** | **t** | **p** |
| --- | --- | --- | --- | --- |
| Intercept | 4.296 (2.042) | 0.295 to 8.298 | 2.1046 | 0.036 |
| PA (MET total) | −0.000140 (0.000020) | −0.000179 to −0.000101 | −6.9559 | <0.001 |
| Sedentary time (min) | 0.023003 (0.000854) | 0.021329 to 0.024677 | 26.9347 | <0.001 |
| BMI | −0.059 (0.053) | −0.163 to 0.045 | −1.1052 | 0.27 |
| Age | −0.013 (0.045) | −0.100 to 0.075 | −0.2807 | 0.779 |
| Education | −0.048 (0.158) | −0.357 to 0.261 | −0.3045 | 0.761 |
| Employment status | 0.082 (0.147) | −0.206 to 0.371 | 0.5594 | 0.576 |
| Household income level | 0.302 (0.177) | −0.046 to 0.649 | 1.7027 | 0.089 |
| Parity | −0.239 (0.253) | −0.736 to 0.257 | −0.9451 | 0.345 |

**Scaled interpretation.** ΔGAD-7 per **+60 min** sedentary = **+1.38** (95% CI **+1.28 to +1.48**); ΔGAD-7 per **+100 MET-min** PA = **−0.014** (95% CI **−0.018 to −0.010**).

**Model diagnostics.** AIC = **2535.81**; BIC = **2581.99**; logLik = **−1256.91**; Residual SE = **2.98** (df = 492); GLS-AR(1) ϕ = **0.290**; all VIF < 2.6.

*Note: MET = metabolic equivalent of task; BMI = body mass index (kg/m²); CI = confidence interval. Unstandardized coefficients. Positive B indicates higher symptom scores.*
