# Supplementary material for: Associations between physical activity and prenatal depression and anxiety symptoms: a cross-sectional study
Source: Front Public Health. 2025 Dec 5;13:1666312. doi: 10.3389/fpubh.2025.1666312 (PMC12714648; doi:10.3389/fpubh.2025.1666312)
Supplement: Supplementary file 3 [file Table_3.docx]

**Supplementary Table 3. Levene’s test for homogeneity of variances across pregnancy trimesters**

| **Outcome** | **F(df1, df2)** | **p-value** | **Conclusion** |
| --- | --- | --- | --- |
| Physical activity (MET-min/week) | F(2, 498) = 0.67 | 0.514 | Homogeneity supported |
| Sedentary time (min/day) | F(2, 498) = 1.12 | 0.329 | Homogeneity supported |
| Depression (CES-D) | F(2, 498) = 0.84 | 0.432 | Homogeneity supported |
| Anxiety (GAD-7) | F(2, 498) = 0.91 | 0.403 | Homogeneity supported |

Levene’s test assessed equality of variances across the three pregnancy trimesters (α=0.05). All tests were non-significant, indicating no evidence against homoscedasticity.

*Note: MET = metabolic equivalent of task; min = minutes.*
